# Antimicrobial resistance and genetic characterization of *Shigella* spp. in Shanxi Province, China, during 2006–2016

**DOI:** 10.1186/s12866-019-1495-6

**Published:** 2019-05-29

**Authors:** Yang Wang, Qiuxia Ma, Ruie Hao, Qiuxiang Zhang, Suxia Yao, Jiting Han, Binzhi Ren, Ting Fan, Limin Chen, Xuebin Xu, Shaofu Qiu, Hongxia Yang

**Affiliations:** 1 Shanxi Province Center for Disease Control and Prevention, Taiyuan, China; 2 Oceanus Plus Medical Development Co., Ltd, Shanghai, China; 3 Shanghai Municipal Center for Disease Control and Prevention, Shanghai, China; 4 Institute of Disease Control and Prevention, PLA, Beijing, China

**Keywords:** Antimicrobial, Resistance, Serotype, MDR, *Shigella*, China

## Abstract

**Background:**

*Shigella* spp., facultative anaerobic bacilli of the family *Enterobacteriaceae*, are one of the most common causes of diarrheal diseases in human worldwide which have become a significant public health burden. So, we aimed to analyze the antimicrobial phenotypes and to elucidate the molecular mechanisms underlying resistance to cephalosporins and fluoroquinolones in *Shigella* isolates from patients with diarrhea in Shanxi Province.

**Results:**

During 2006–2016, we isolated a total of 474 *Shigella* strains (including 337 *S. flexneri* and 137 *S. sonnei*). The isolates showed high rates of resistance to traditional antimicrobials, and 26, 18.1 and 3.0% of them exhibited resistance to cephalosporins, fluoroquinolones and co-resistance to cephalosporins and fluoroquinolones, respectively. Notably, 91.1% of these isolates, including 22 isolates that showed an ACTSuT profile, exhibited multidrug resistance (MDR). The resistance rates to cephalosporins in *S. sonnei* isolates were higher than those in *S. flexneri.* Conversely, the resistance rates to fluoroquinolones were considerably higher in *S. flexneri* isolates. Among the 123 cephalosporins-resistant isolates, the most common extended-spectrum beta-lactamase gene was *bla*_TEM-1_, followed by *bla*_CTX-M_, *bla*_OXA-1_, and *bla*_SHV-12_. Six subtypes of *bla*_CTX-M_ were identified, *bla*_CTX-M-14_ (*n* = 36) and *bla*_CTX-M-55_ (*n* = 26) were found to be dominant. Of all the 86 isolates with resistance to fluoroquinolones and having at least one mutation (Ser83Leu, His211Tyr, or Asp87Gly) in the the quinolone resistance-determining regions of *gyr*A, 79 also had mutation of *par*C (Ser80Ile), whereas 7 contained plasmid-mediated quinolone resistance genes including *qnr*A, *qnr*B, *qnr*S, and *aac(60)-Ib-cr.* Furthermore, pulsed-field gel electrophoresis analysis (PFGE) showed a considerable genetic diversity in *S. flexneri* isolates. However, the *S. sonnei* isolates had a high genetic similarity.

**Conclusions:**

Coexistence of diverse resistance genes causing the emergence and transmission of MDR might render the treatment of shigellosis difficult. Therefore, continuous surveillance might be needed to understand the actual disease burden and provide guidance for shigellosis.

**Electronic supplementary material:**

The online version of this article (10.1186/s12866-019-1495-6) contains supplementary material, which is available to authorized users.

## Background

*Shigella* spp., facultative anaerobic bacilli of the family *Enterobacteriaceae*, are one of the most common causes of diarrheal diseases in human worldwide and have become a significant public health burden [1]. Globally, nearly 167 million *Shigella* episodes per year are estimated, of which 99% are reported in developing countries. It is reported that almost 61% of all deaths attributed to shigellosis are in children under 5 years old [2]. In China, nearly half a million shigellosis cases are reported every year, which is situated at the top four notifiable infectious disease [2].

Researchers have classified the genus *Shigella* into 4 serogroups: *S. dysenteriae, S. flexneri, S. boydii, and S. sonnei* based on biochemical and serological properties. The *S. flexneri* is the predominant species in developing countries [1], where is in poor sanitation, such as in mainland China. Otherwise, the *S. sonnei* is mainly found in industrialized countries [2, 3], and has been implicated in source outbreaks [4]. However, in some Asian countries and some developed regions of China, *S. sonnei* is gradually overtaking *S. flexneri* as the main pathogenic bacteria that cause shigellosis [5–8].

Based on the national surveillance data from 2009, the annual shigellosis-related morbidity rate was 20.3 cases per 100,000 people in China, and the two major causative species were *S. flexneri* and *S. sonnei* [9]. To date, at least 20 *S. flexneri* serotypes have been recognized and reported, such as 1a, 1b, 1c (or 7a), 1d, 2a, 2b, 2v, 3a, 3b, 4a, 4av, 4b, 5a, 5b, X, Xv, Y, Yv, 6, and 7b [10, 11]. Some new serotypes such 4 s and 2 variants have been found and disseminated in China, likely leading to a serious threat to public health security [11, 12]. In some developing countries, *S. flexneri* 1b is the most commonly encountered serotypes, followed by *S. flexneri* 2a [1].

Infants, the elderly, and immunocompromised individuals with *Shigella* infection require antimicrobial treatment to shorten the clinical symptom duration and carriage and reduce the spread of infection [13]. The World Health Organization recommends fluoroquinolones and cephalosporins as the preferred drugs for the treatment of *Shigella* infections. With the extensive use of these antimicrobials, antimicrobial resistance is increasing remarkably in *Shigella* isolates. Since the first report of norfloxacin-resistant *Shigella* in 1949 in Japan [14], increasing number of *Shigella* isolates with multiple drug resistance (MDR, defined as resistance to three or more classes of antimicrobials) has been discovered in the world. It was reported that some factors could influence the antimicrobial susceptibility patterns of *Shigella* isolates, such as the geographic location, year, antimicrobial use, and antimicrobial agents [15]. However, few studies have investigated the antimicrobial resistance of *Shigella* in different cities of China, such as Shanghai and Beijing [5, 6].

Selection of the most effective antimicrobial agents for shigellosis treatment requires the understanding of the antimicrobial susceptibility profiles of prevalent strains [16]. This study aimed to analyze the antimicrobial resistance profiles of *Shigella* isolates from Shanxi Province during 2006 to 2016, and to elucidate the molecular mechanisms underlying the emergence of MDR in these isolates.

## Results

### Bacterial isolates, serotyping, and biochemical characterization

**Fig. 1:**
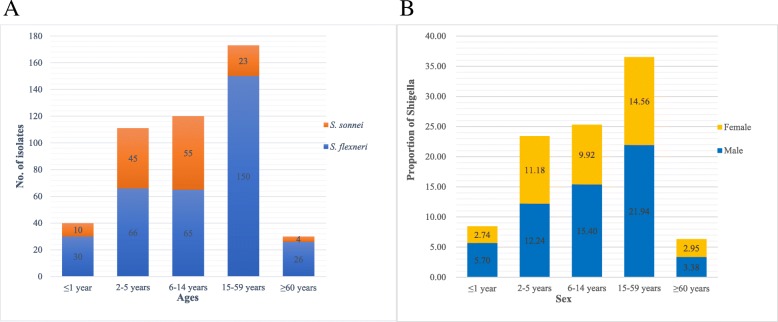
Distribution of human *Shigella* isolates in patients by (**a**) species and (**b**) sex of all age groups in Shanxi Province, China

**Fig. 2:**
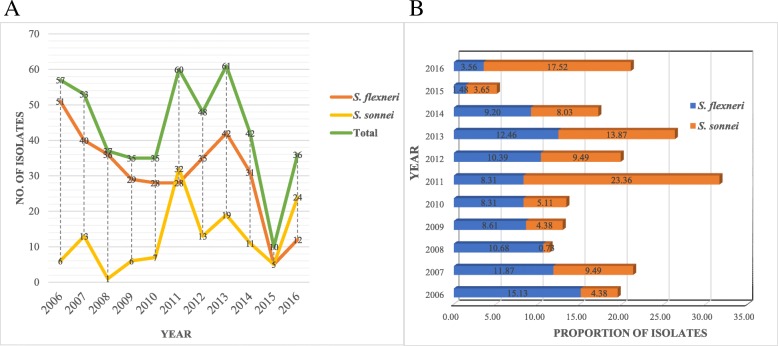
Distribution of human *Shigella* isolates in patients during 2006 and 2016 in Shanxi Province, China. **a**: No. of isolates (A total of 474 *Shigella* strains, including 337 *S. flexneri* and 137 *S. sonnei* strains); **b**: proportion of isolates

**Fig. 3:**
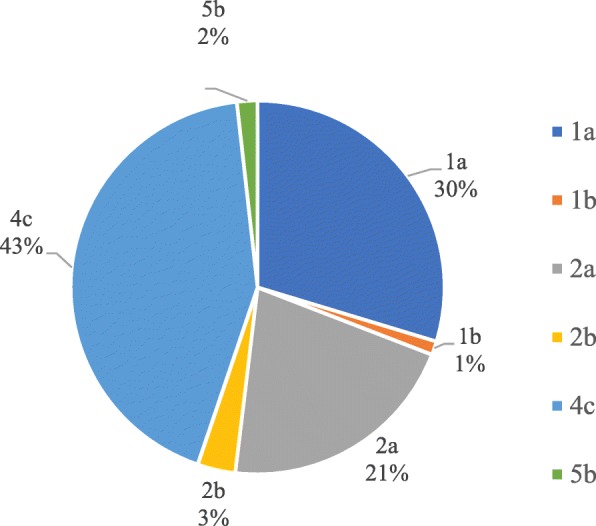
The serotypes of *S. flexneri* species from 2006 to 2016 in Shanxi Province, China

During our 11-year routine surveillance (from 2006 and 2016) of shigellosis, a total of 474 *Shigella* isolates, including 337 *S. flexneri* (71.1%) strains and 137 *S. sonnei* (28.9%) isolates, but no *S. dysenteriae* and *S. boydii* were identified from patients with diarrhea in Shanxi Province. The age of the patients ranged from 2 months to 87 years (Fig. 1). The patients aged 15–59 years accounted for the highest proportion of 36.5% of all age groups (*n* = 173), whereas patients over 60 years old were the least susceptible, with a proportion of 6.3% (*n* = 30). Among all age groups, the proportion of males was higher than that of females (Fig. 1b). The male to female ratio for the patients was 1.42:1. Among the *Shigella* isolates, the constituent ratio of *S. flexneri* was higher than that of *S. sonnei* isolates every year, except in 2011 and 2016 (Fig. 2). Several *S. flexneri* serotypes were found in the 337 *S. flexneri* isolates, including serotypes 1a, 1b, 2a, 2b, 4c, and 5b. Notably, serotypes 4c and 1a were the main serotypes, accounting for 43 and 30%, respectively (Fig. 3). These results suggested that *S. sonnei* and *S. flexneri* are the prevalent species in Shanxi Province of China, especially the *S. flexneri* serotypes 4c and 1a.

### Antimicrobial susceptibility testing

Among the 474 isolates, only 2 (0.4%) were susceptible to all 21 antimicrobials. Resistance to ampicillin was the most common (97.7%), followed by that to ticarcillin (94.9%), trimethoprim/sulfamethoxazole (88.4%), tetracycline (78.3%), chloramphenicol (57.4%), gentamicin (40.5%), cefazolin (26.2%), ceftriaxone (26.0%), norfloxacin (18.1%), cefoperazone (17.9%), piperacillin (16.0%), tobramycin (8.9%), aztreonam (5.7%), levofloxacin (2.3%), ticarcillin/clavulanic acid (1.7%), imipenem (0.8%), ceftazidime (0.6%), cefoxitin and amikacin (0.2%). None of the isolates was resistant to cefepime and nitrofurantoin (Table 1). The antibiotic resistance rates differed between *S. sonnei* and *S. flexneri.* The resistance rates of *S. flexneri* isolates to the top three antibiotics ampicillin, ticarcillin, and trimethoprim/sulfamethoxazole were 98.5, 95.0, and 85.2%, respectively. However, the resistance rate to trimethoprim/sulfamethoxazole (96.4%) was the highest in *S. sonnei*, which was considerably higher than that of *S. flexneri,* followed by that to ampicillin (95.6%) and ticarcillin (94.9%). Further, the resistance rates for tetracycline, gentamicin, and piperacillin were considerably higher in *S. sonnei* isolates, especially to cephalosporins, such as cefazolin, ceftriaxone, cefoperazone, and ceftazidime (*P* < 0.05). However, the resistance rates for chloramphenicol, norfloxacin, and levofloxacin in *S. flexneri* were considerably higher than those in *S. sonnei* isolates (*P* < 0.05). (Table 1). None of the *S. sonnei* isolates was resistance to cefoxitin and amikacin; the *S. flexneri* isolates also showed a low resistance rate (0.3%) to both the antibiotics. More importantly, 14 (3.0%) isolates showed co-resistance to third-generation cephalosporins and fluoroquinolones.

**Table 1:** Comparison of antimicrobial resistance to 21 antibiotics among *Shigella* strains from Shanxi Province, China

Antibiotic	*S. flexneri*			*S. sonnei*			Total			*χ* ^2^	*P* value
	No. Tested	No. Resistant	(%)	No. Tested	No. Resistant	(%)	No. Tested	No. Resistant	(%)		
AMP	337	332	98.5	137	131	95.6	474	463	97.7	3.6	0.06
PIP	337	44	13.1	137	32	23.4	474	76	16.0	7.7	0.01
TIC	337	320	95.0	137	130	94.9	474	450	94.9	0.0	0.98
CFZ	337	67	19.9	137	57	41.6	474	124	26.2	23.8	0.00
FOX	337	1	0.3	137	0	0.0	474	1	0.2		
CRO	337	68	20.2	137	55	40.2	474	123	26.0	20.2	0.00
CAZ	337	1	0.3	137	2	1.5	474	3	0.6	2.1	0.20
CFP	337	47	14.0	137	38	27.7	474	85	17.9	12.6	0.00
FEP	337	0	0.0	137	0	0.0	474	0	0.0		
NOR	337	84	24.9	137	2	1.5	474	86	18.1	31.6	0.00
LEV	337	11	3.3	137	0	0.0	474	11	2.3	4.6	0.04
IMP	337	3	0.9	137	1	0.7	474	4	0.8	0.0	1.00
GEN	337	82	24.3	137	110	80.3	474	192	40.5	166.9	0.00
TO	337	29	8.6	137	13	9.5	474	42	8.9	0.1	1.00
AK	337	1	0.3	137	0	0.0	474	1	0.2		
SXT	337	287	85.2	137	132	96.4	474	419	88.4	105.3	0.00
TE	337	248	73.6	137	123	89.8	474	371	78.3	15.0	0.00
C	337	265	78.6	137	7	5.1	474	272	57.4	215.3	0.00
ATM	337	17	5.0	137	10	7.3	474	27	5.7	7.1	0.01
NIT	337	0	0.0	137	0	0.0	474	0	0.0		
TIM	337	5	1.5	137	3	2.2	474	8	1.7	0.2	0.70

A *p* value that < 0.05 was considered statistically significant that compared the resistance rates between *S. flexneri* and *S. sonnei*

Moreover, notable differences were noted in antibiotic resistance profiles especially those changed significantly during 2006–2011 and 2012–2016. Of all the *Shigella* strains, the resistance rate to cefazolin, ceftriaxone, norfloxacin, and aztreonam was 23.8, 24.2, 15.5, and 1.8%, respectively, during 2006–2011, and increased to 29.4, 28.4, 26.9, and 11.2% during 2012–2016. Conversely, the resistance rate to piperacillin, tobramycin, Trimethoprim/sulfamethoxazole, tetracycline, and chloramphenicol was 18.8, 12.3, 90.3, 85.6, and 64.6%, respectively, during 2006–2011 and decreased to 12.2%. 4.1, 85.8, 68.0, and 47.2% during 2012–2016 (Table 2).

**Table 2:** Resistance to antibiotic of all *Shigella* isolates from Shanxi Province during 2006–2011 and 2012–2016

Antibiotic	2006–2011			2012–2016			*χ* ^2^	*P* value
	No. Tested	No. Resistant	(%)	No. Tested	No. Resistant	(%)		
AMP	277	272	98.2	197	191	97.0	0.8	0.54
PIP	277	52	18.8	197	24	12.2	54.5	0.00
TIC	277	266	96.0	197	184	93.4	1.7	0.20
CFZ	277	66	23.8	197	58	29.4	1.9	0.17
FOX	277	0	0	197	1	0.5	–	–
CRO	277	67	24.2	197	56	28.4	1.1	0.30
CAZ	277	1	0.4	197	2	1.0	0.8	0.57
CFP	277	48	17.3	197	37	18.8	0.2	0.68
FEP	277	0	0	197	0	0	–	–
NOR	277	43	15.5	197	53	26.9	6.7	0.01
LEV	277	9	3.3	197	2	1.0	1.3	0.34
IMP	277	2	0.7	197	2	1.0	0.1	1.00
GEN	277	111	40.1	197	81	41.1	7.0	0.01
TO	277	34	12.3	197	8	4.1	9.6	0.00
AK	277	0	0	197	1	0.5	–	–
SXT	277	250	90.3	197	169	85.8	2.2	0.14
TE	277	237	85.6	197	134	68.0	0.6	0.43
C	277	179	64.6	197	93	47.2	1.1	0.30
ATM	277	5	1.8	197	22	11.2	34.0	0.00
NIT	277	0	0	197	0	0	–	–
TIM	277	3	1.1	197	5	2.5	1.8	0.27

Further, MDR was observed in 91.1% (*n* = 432) of the isolates, of which 91.1, 70.7, and 24.9% were resistant to ≥3, ≥ 4, and ≥ 5 CLSI classes of antimicrobials were found in, respectively (Table 3). Among the MDR isolates, 412 (86.5%), 242 (50.1%), and 22 (4.6%) isolates showed an AT/S (defined as resistance to ampicillin and trimethoprim/sulfamethoxazole), ACT/S (defined as resistance to ampicillin, chloramphenicol, and trimethoprim/sulfamethoxazole) and ACTSuT resistance pattern (defined as resistance to ampicillin, chloramphenicol, tobramycin, trimethoprim/sulfamethoxazole and tetracycline), respectively.

**Table 3:** Antibiotic resistance patterns of *Shigella* from 2006 to 2016 in Shanxi province, China

MDR patterns	*S. flexneri*			*S. sonnei*			*χ* ^2^	*P* value
	No. Tested	No. Resistant	(%)	No. Tested	No. Resistant	(%)		
No resistance detected	337	0	0	137	2	1.5	–	–
Resistance ≥1 CLSI class	337	337	100	137	135	98.5	–	–
Resistance ≥2 CLSI classes	337	327	97.0	137	133	97.1	–	–
Resistance ≥3 CLSI classes	337	302	89.6	137	130	94.9	3.4	0.07
Resistance ≥4 CLSI classes	337	231	68.6	137	104	75.9	2.6	0.11
Resistance ≥5 CLSI classes	337	84	24.9	137	34	24.8	18.0	0
AT/S	337	284	84.3	137	128	93.4	7.2	0.007
ACT/S	337	237	70.3	137	7	5.1	5.1	0.048
ACTSuT	337	22	6.5	137	0		9.4	0.002

1 CLSI class: means resistance to one third-generation cephalosporin or one quinolone

2 CLSI classes: means resistance to two third-generation cephalosporin or two quinolones, or one third-generation cephalosporin and one quinolone

3 CLSI classes: means resistance to one third-generation cephalosporin and two quinolones, or one third-generation cephalosporin and two quinolones

4 CLSI classes: means resistance to two third-generation cephalosporins and two quinolones

5 CLSI classes: means resistance to three third-generation cephalosporins and two quinolones

We have added these under the Table 3 in our manuscript

AT/S: resistance to ampicillin, trimethoprim-sulfamethoxazole

ACT/S: resistance to ampicillin, chloramphenicol, trimethoprim-sulfamethoxazole

ACTSuT: resistance to ampicillin, chloramphenicol, tobramycin, Trimethoprim/ sulfamethoxazole and tetracycline

### Molecular analysis of antibiotic-resistant determinants and integrons

**Fig. 4:**
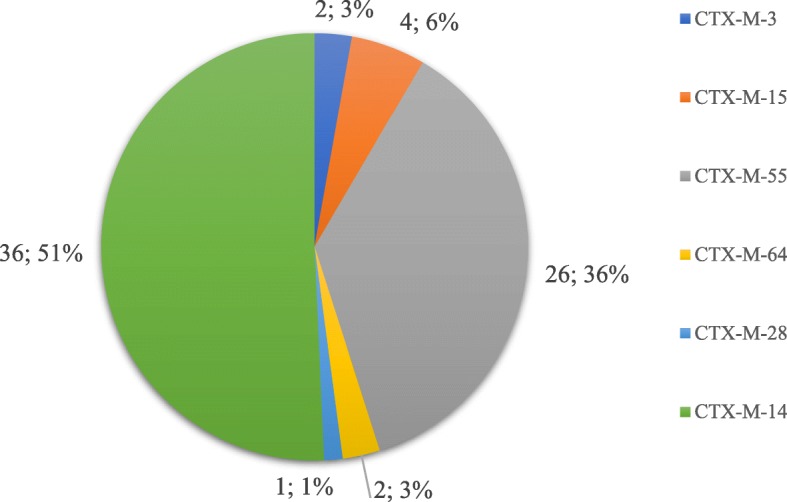
The CTX-M proportions of cephalosporin-resistant *Shigella* isolates in Shanxi Province. The digit before the semicolon; indicates the number of isolates containing *bla*_CTX-M_ genes, and the digit after the semicolon indicates the percentage of isolates containing *bla*_CTX-M_ genes

A total of 195 *Shigella* isolates (including 109 cephalosporin-resistant isolates, 72 quinolone-resistant isolates, and 14 co-resistance isolates) were tested for the presence of antimicrobial resistance determinants and integrons. PCR results showed that all 195 tested isolates were negative for *bla*_VIM_ and *bla*_NDM_, but positive for *bl*a_SHV,_ *bla*_TEM_, *bla*_OXA_, *bla*_CTX-M_, *intI*1 and *intI*2 gene regions (Table 4). Further, 90 (73.2%, *n* = 123) isolates harbored *bla*_TEM_, and sequencing results of *bla*_TEM_ showed 100% identity with *bla*_TEM-1_. Moreover, 71 isolates were positive for *bla*_CTX-M_, of which 36 isolates harbored *bla*_CTX-M-14_, 4 harbored *bla*_CTX-M-15_, 26 (21.1%, *n* = 123) harbored *bla*_CTX-M-55_, 2 isolates harbored *bla*_CTX-M-28_ and *bla*_CTX-M-64_ each, and only one isolate simultaneously harbored both *bla*_CTX-M-3_ and *bla*_CTX-M-14_ (Fig. 4). Forty-nine isolates harbored *bla*_OXA-1_, with a positive rate of 39.8%. Eighteen strains were positive for *bla*_SHV_. Among the tested isolates, 139 and 167 isolates contained class 1 and class 2 integrons, respectively. Class 1 integrons positive isolates includ 16 *S. sonnei* isolates and 123 *S. flexneri* isolates, therefore class 2 positive isolates include 47 *S. sonnei* strains and 120 *S. flexneri* strains. All class 1 integrons harbored *bla*_OXA-1_, which is present on the Tn2603 transposons [17] and *aadA1* gene cassettes, whereas class 2 integrons are included in *dfrA1, sat1*, and *aadA1* gene cassettes.

**Table 4:** Genetic determinants for cephalosporins and quinolones resistance in *Shigella* strains

Content	*S. flexneri*			*S. soneni*			Total			*χ* ^2^	*P* value
	No. Tested	No. Resistant	Rate(%)	No. Tested	No. Resistant	Rate(%)	No. Tested	No. Resistant	Rate(%)		
Cephalosporins	337	68	20.2	137	55	40.1	474	57	12.0	20.2	0
Quinolones	337	85	25.2	137	2	1.5	474	70	14.8	36.7	0
*bla*_CTX-M-1-group_	68	16	23.5	55	19	34.5	123	35	28.5	0.2	0.23
*bla*_CTX-M-9-group_	68	12	17.6	55	24	43.6	123	36	29.3	9.9	0
*bla*_OXA_	68	44	64.7	55	5	9.1	123	49	39.8	39.2	0
*bla*_TEM_	68	30	44.1	55	5	9.1	123	35	28.5	18.3	0
*bla*_SHV_	68	5	7.4	55	0	0.0	123	5	4.1	1.1	0.49
*bla*_CMY_	68	13	19.1	55	0	0.0	123	13	10.6	11.8	0

A *p* value that < 0.05 was considered statistically significant that compared the resistance rates between *S. flexneri* and *S. sonnei*

Among the 86 quinolone-resistant isolates, no point mutations were noted in the QRDRs of *gyr*B and *par*E, but point mutations were noted in *gyr*A and *par*C in the most resistant isolates. All the 86 quinolone-resistant isolates had the *gyr*A mutation of Ser83Leu and His211Tyr; 7 isolates had the *gyr*A mutation of Asp87Gly; and 79 had the *par*C mutation of Ser80Ile. Seven isolates were positive for *qnr*A, *qnr*B and *acc(6′)-Ib-cr*. Forty-five (52.3%) isolates were positive for *qnr*S.

Notably, among the 14 isolates that concurrently exhibited reduced susceptibility to cephalosporins and quinolone, 13 contained ESBL and PMQR genes. Six isolates contained four types of antimicrobial-resistant genes: *bla*_CTX-M-55_/*bla*_OXA_/*bla*_TEM_/*qnr*S (*n* = 2) and *bla*_CTX-M-55_/*bla*_OXA_/*bla*_TEM_/ *qnr*B (*n* = 1), *bla*_OXA_/*bla*_TEM_/*bla*_CMY_/*qnr*S (n = 1), *bla*_CTX-M-14_/*bl*_aOXA_/*bla*_TEM_/*qnr*S (n = 1), and *bla*_CTX-M-14_/*bla*_TEM_/*qnr*S/*acc (6′)-Ib-cr* (n = 1). Five isolates contained three types of genes: *bla*_CTX-M-3_/*qnr*S/ *acc (6′)-Ib-cr*, *bla*_OXA_/*bla*_TEM_/*qnr*S, *bla*_CTX-M-15_*/bla*_OXA_/bla_SHV_, *bla*_CTX-M-14_/*bla*_TEM_/*qnr*S, and *bla*_CTX-M-55_/*bla*_TEM_/*qnr*S (all n = 1). Two isolates had two types of genes: *bla*_CTX-M-14_/*bla*_TEM_ and *bla*_TEM_/*qnr*S (n = 1 each). Only one isolate did not have any resistance genes (Table 5).

**Table 5:** Genetic determinants for cephalosporins and quinolones resistance in *Shigella* strains showing concurrently decreased susceptibility to these antibiotics

Strains	Resistance Phenotypes						QRDR mutation		PMQR	ESBLs
	CAZ	CRO	CFP	CFZ	LEV	NOR	gyrA	parC		
LJ-07-011	S	R	R	R	R	R	Ser83Leu, His211Tyr	Ser80Ile	*qnr*S	*bla*_OXA_,*bla*_TEM_
LJ-07-037	S	R	R	R	R	R	Ser83Leu, His211Tyr	Ser80Ile	*qnr*S, *acc(6′)-Ib-cr*	*bla*_CTX-M-3_
LJ-07-042	S	R	R	R	R	R	Ser83Leu, His211Tyr	Ser80Ile	*qnr*S	b*la*_OXA_,*bla*_TEM_,*bla*_CMY_
LJ-12-049	I	R	R	R	S	R	Ser83Leu, His211Tyr	Ser80Ile		
LJ-13-009	I	R	R	R	I	R	Ser83Leu, His211Tyr	Ser80Ile	*qnr*S	*bla*_CTX-M-55_, *bla*_OXA,_ *bla*_TEM_
LJ-13-010	I	R	R	R	I	R	Ser83Leu, His211Tyr	Ser80Ile	*qnr*S	*bla*_CTX-M-55_, *bla*_OXA,_ *bla*_TEM_
LJ-13-011	S	R	R	R	I	R	Ser83Leu, His211Tyr	Ser80Ile	*qnrS, acc(6′)-Ib-cr*	*bla*_CTX-M-14_,*bla*_TEM_
LJ-13-040	I	R	R	R	I	R	Ser83Leu, His211Tyr	Ser80Ile		*bla*_CTX-M-15_,*bla*_OXA_,*bla*_SHV_
LJ-14-015	I	R	R	R	I	R	Ser83Leu, His211Tyr	Ser80Ile	*qnr*B	*bla*_CTX-M-55,_ *bla*_OXA,_ *bla*_TEM_
ZD-13-001	S	R	I	R	S	R	Ser83Leu, His211Tyr	Ser80Ile	*qnr*S	*bla*_TEM_
ZD-13-022	S	R	R	R	S	R	Ser83Leu, His211Tyr	Ser80Ile		*bla*_CTX-M-14_,*bla*_TEM_
ZD-14-029	S	R	I	R	I	R	Ser83Leu, His211Tyr	Ser80Ile	*qnr*S	*bla*_CTX-M-14_,*bla*_TEM_,*bla*_OXA_
ZD-15-020	S	R	R	R	S	R	Ser83Leu, sp87Gly, His211Tyr	Ser80Ile	*qnr*S	*bla*_CTX-M-14_,*bla*_TEM_
ZD-16-027	I	R	R	R	S	R	Ser83Leu, His211Tyr	Ser80Ile	*qnr*S	*bla*_CTX-M-55_,*bla*_TEM_

*R* Resistance, *I* Intermediate resistance, *S* Sensitive

### PFGE analysis

**Fig. 5:**
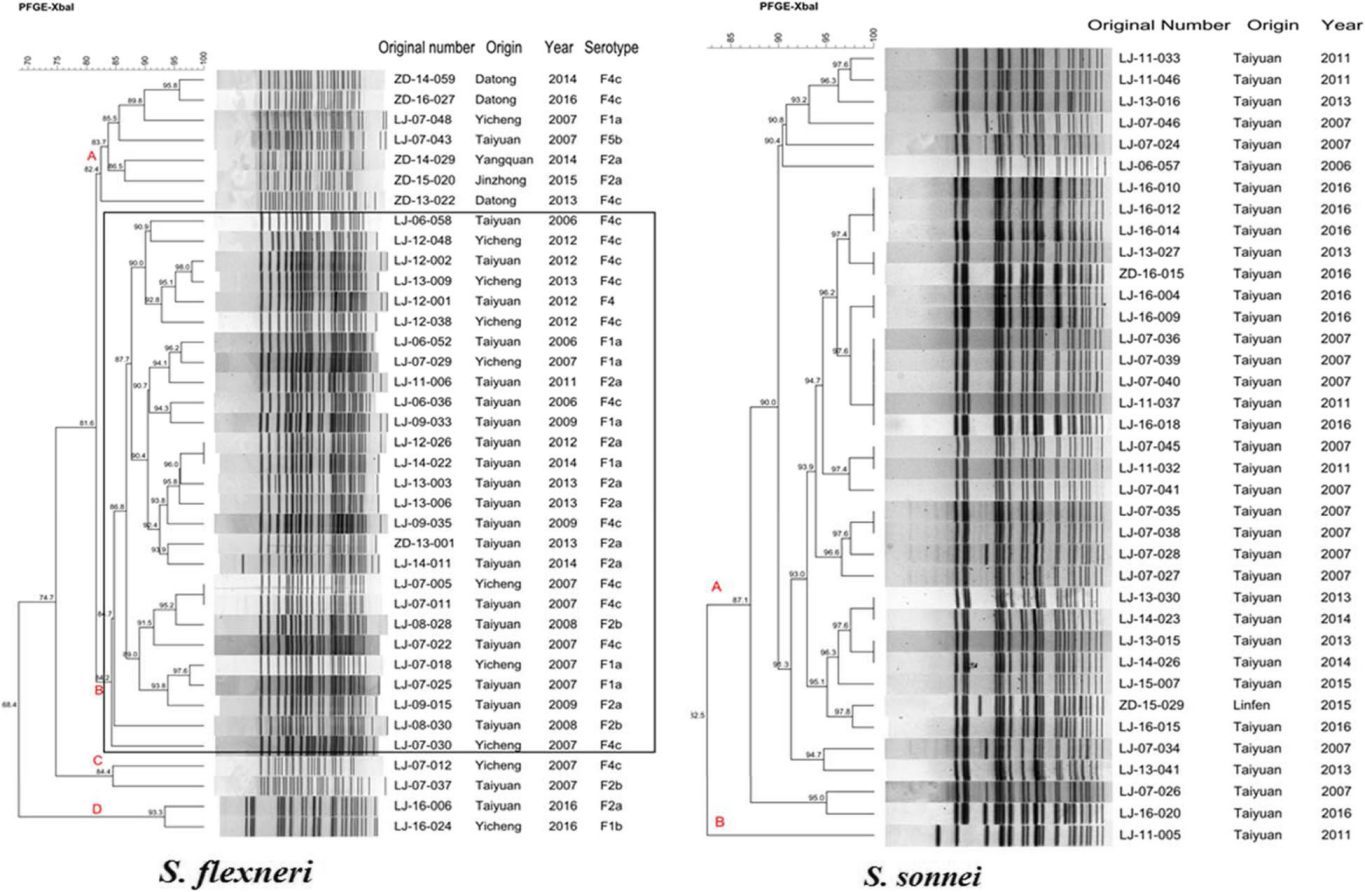
Pulsed-field gel electrophoresis dendrogram of *Shigella* spp. during 2006 and 2016 in Shanxi Province, China. The original number, origin, serotype, and isolation year are indicated for each strain

PFGE was performed to determine the genetic relatedness among the 75 randomly selected *Shigella* isolates from different years and regions in Shanxi Province. The results of PFGE suggested that the 38 *S. flexneri* isolates generated 36 PFGE patterns (Fig. 5 a). All isolates could be categorized into four distinct groups (A-D) with a similarity of approximately 82%, including F1a, F2a, F2b, F4c, and F5b serotypes. This suggests considerable genetic diversity among the 38 *S. flexneri* isolates between different regions and years in Shanxi Province. Notably, group B was the major PFGE type of *S. flexneri* in Shanxi. Conversely, the 37 *S. sonnei* strains generated 25 PFGE patterns (Fig. 5 b), but formed a single cluster except one isolate (LJ-11-005) with a similarity of 82%. This result suggested that the *S. sonnei* strains had high genetically similarity in Shanxi Province.

## Discussion

The emergence of novel and atypical bacterial serotypes in nature is attributed to serotype conversion, which often occurs in response to the protective host immune response [18]. Since the 1990s, several new *S. flexneri serotypes* (e.g., 1c and SFxv) have emerged and become the most prevalent ones in some countries [19]. SFxv, first appeared in Henan Province, China, in 2001 and was considered one of the predominant serotypes in Shanxi, Gansu, and Anhui Provinces from 2002 to 2006 [19, 20]. The prevalence and characterization of human *Shigella* infections in Henan Province, China were determined in 2006 [20]. Data on the prevalence of *S. flexneri* serotypes causing shigellosis in mainland China from 2001 to 2010 suggest that SFxv is the second most predominant serotype after 2a [21]. However, our results showed that the top three common *Shigella* serotypes in Shanxi Province were *S. sonnei*, *S. flexneri* serotypes 4c and 1a, which differed from those reported previously [18–21]. Interestingly, our data indicated that *S. sonnei* has replaced *S. flexneri* as the predominant species causing shigellosis in Shanxi Province, which was consistent with the findings of previous studies [5, 6]. The increasing of proportion of *S. sonnei* is related to regional economic development and sanitary conditions. Shanxi is a developing and mountainous province with poor sanitary, which could promote the increasing of *S. sonnei* strains. Furthermore, it could also be conducive to the prevalence and dissemination of *S. sonnei* strains with MDR.

In our study, *S. flexneri* tended to gradually increase and reach a peak in 2011, and then slowly decline again, whereas *S. sonnei* showed an opposite tendency. These trends and patterns were similar with those noted in developed countries [22]. The increasing antimicrobial resistance of *Shigella* species is a major problem in the treatment of *Shigella* gastroenteritis, especially of the MDR *Shigella* strains. Approximately 91.1% of the strains in our study showed MDR profiles, which is significantly higher than the rate of 41.6% (1762/4234) from the NARMS report (2005~2014) [23]. All the MDR strains were highly resistant to the traditional antimicrobials such as ampicillin, ticarcillin, trimethoprim/sulfamethoxazole, and tetracycline. One of the reasons for the rapid accumulation of resistance has been reported to be the excessive or inappropriate use of antibiotics in outpatients in China [24, 25].

Fluoroquinolones and third-generation cephalosporins are the recommended first-line and alternatives drugs by the World Health Organization for empiric shigellosis treatment [26]. Our study further indicated that the current resistance patterns have changed, and empirical therapy should be modified in accordance with these changes. Thus, the treatment should be based on the susceptibility patterns and antimicrobials with current resistance might become effective in the future.

Moreover, in our study, 26.2% of cephalosporins-resistant *Shigella* isolates were found, which was considerably higher than the rate indicated in the NARMS report (lower than 1% from 2005 to 2014). The *S. sonnei* isolates showed higher resistance rates to cephalosporins, whereas the *S. flexneri* isolates had higher level resistance to fluoroquinolones. More importantly, we found 14 MDR isolates with co-resistance to fluoroquinolones and cephalosporins. If these MDR strains are prevalent worldwide, it might become a remarkable global public health concern. Our findings indicated that continuing monitor the antimicrobial resistance of *Shigella* isolates is necessary to help determine the appropriate antimicrobial therapy for patients with *Shigella* infection. More importantly, determining the mechanisms of antimicrobial resistance is necessary to assist in developing measures to prevent antibiotic resistance.

The increasing antibiotic resistance and rate led us to investigate the genetics and mechanisms of antibiotic resistance. Under the influence of various antibiotics, bacteria have a strong ability to obtain resistance genes for survival. Class 1 and class 2 integrons, which contain resistance genes and can be coordinately excised or integrated [2], might account for the horizontal transfer of resistance genes. In our study, 71.3% (*n* = 139) and 85.6% (*n* = 167) of isolates harbored class 1 and class 2 integrons, followed by the *bla*_OXA-1_ + *aadA1* and *dfrA1 + sat1 + aadA1* gene cassettes, conferring resistance to trimethoprim and streptomycin [27].

In addition, of the 123 cephalosporin-resistant isolates, 73.2% harbored the *bla*_TEM-1_ resistance gene, 57.7% harbored *bla*_CTX-M_, most of which were *bla*_CTX-M-14_, followed by *bla*_CTX-M-55_, *bla*_CTX-M-15_, *bla*_CTX-M-28,_ and *bla*_CTX-M-64_. Further, 39.8% harbored *bla*_OXA_, and 14.6% harbored *bla*_SHV_. The *bla*_TEM-1_ gene exists at high frequencies in antibiotic-resistance bacteria and often confers resistance to penicillin and other *β*-lactamic antibiotics [28]. whereas the OXA-type *β*-lactamic, with high hydrolytic activity against oxacillin and cloxacillin often confer resistance to ampicillin and cephalothin [29]. Sequencing analysis showed that all the *bla*_OXA_ genes were *bla*_OXA-1_, which is consistent with the findings of a previous study on *Shigella* strains [30]. Plasmid-mediated transfer of different *bla*_CTX-M_ genes was thought to be the reason for introduction of these genes into the isolates at different times [25], indicating that *bla*_CTX-M-14_ and *bla*_CTX-M-55_ genes might have long been circulating among *Shigella* isolates in Shanxi Province.

PMQR was initially identified in *Klebsiella pneumoniae* in 1998 [31]; since then, various types of PMQR genes have been detected worldwide. Quinolone levels and/or fluoroquinolone resistance have been mostly attributed to mutations in the target enzymes gyrase (*gyrA* and *gyrB*) and topoisomerase IV (*parC* and *parE*), and the presence of plasmid-borne mechanisms owing to the proteins encoded by *qnrA*, *qnrB*, *qnrS* and *aac*(*6*′)*-Ib-cr* [32, 33]. The mutations of *gyr*A and *par*C at positions 67–106 are known to be the predominant mutations that can lead to fluoroquinolone resistance [34]. The *gyrA* Ser83Leu is the most frequently observed in *Shigella* species, and usually results in high-level resistance to the first-generation quinolone nalidixic acid [35]. The presence of additional mutations of *gyrA* (Asp87Gly/Asn and His211Tyr) and *par*C (Ser80Ile) results in resistance to fluoroquinolones [36]. Moreover, the mutation of His211Tyr in *gyr*A is very common in fluoroquinolone-resistant *Shigella* [30]*.* In our study, 100% of the quinolone-resistant *Shigella* isolates had point mutations in *gyr*A (Ser83Leu, Asp87Gly/Asn, and His211Tyr) and *par*C (Ser80Ile). *S. flexneri* serotypes (such as 1a, 2a, 2b, and 4c) carrying the *qnrS* gene have been globally reported with low incidence [37, 38]. In our study, 45 (52.3%) of the strains contained *qnrS*, of which 11 showed high resistance to levofloxacin and norfloxacin. The *aac(6*′*)-Ib-cr* is reported to be responsible for low-level resistance to fluoroquinolones [39] and was first isolated from *Shigella* strains in 1998 [38]. Moreover, seven of the quinolone-resistant isolates were *aac(6*′*)-Ib-cr*–positive, suggesting that the *qnrS* and *aac(6*′*)-Ib-cr* genes had long been present in Shanxi Province. The *qnr*A and *qnr*B were reported to be located on plasmids carrying *bla* genes (such as *bla*_SHV_ and *bla*_CTX_) [40]. In this study, seven strains also contained *qnr*A and *qnr*B each, and the *qnr*B-positive isolate coharbored *bla*_CTX-M-55,_ *bla*_OXA_, and *bla*_TEM_. Our results are consistent with those of previous studies and the theory suggesting quinolone resistance determinants alone might have a weak effect on resistance levels; however, when combined with other determinants, resistance can be obtained [41]. The various resistance genes facilitate the dissemination of resistance determinants and the survival of bacteria under the selective pressure of various antibiotics.

Besides, in our study, the PFGE dendrogram showed that the *S. sonnei* isolates are closely related (82% similarity), indicating that they are possibly derived from a common parental strain. In contrast, the *S. flexneri* isolates (including F1a, F2a, F2b, F4c, and F5b serotypes) showed lower degrees of similarity, suggesting that they are likely derived from diverse sources, such as from different years, sources or origins. And the group B PFGE pattern was the major PFGE type of *S. flexneri* in Shanxi Province. Although PFGE has high concordance with epidemiological and genetical relatedness, and is considered as the “gold standard” fingerprinting method used for the discrimination and identification within PulseNet, it might not be effective in some *Shigella* or *Salmonella* species, which warrants further investigation with complementary molecular tools as multilocus sequence typing (MLST) [42].

## Conclusions

In summary, we reported the distribution of *Shigella* serotypes and analyzed the common occurrence of MDR and resistance mechanisms in *Shigella* isolates in Shanxi Province during 2006 and 2016, China. The diverse antimicrobial resistance patterns and multi-types resistance genes were observed. Future studies should be focused on identifying ways to prevent the dissemination of these antimicrobial-resistance genes. Our data might provide a strategy for the treatment of infections caused by *Shigella* strains in Shanxi Province, China. Therefore, continuous surveillance might be imperative to determine the distribution and resistance development of *Shigella*, and to understand the actual disease burden and provide guidance for the clinical treatment of shigellosis. Furthermore, without treatment of shigellosis, especially caused the MDR *Shigella*, it might become a dominant strain and be prevalent in Shanxi Province, and spread worldwide, leading to the outbreaks of *Shigella* and causing significant public health and disease burden.

## Materials

### Bacterial isolates, serotyping, and biochemical characterization

All the *Shigella* strains were isolated from fresh fecal samples, which were collected from outpatients with diarrhea or dysentery in four sentinel hospitals and two regional Centers for Disease Control and Prevention (one in Taiyuan City and the other in Yicheng County) in Shanxi Province based on a provincial pathogen monitoring system. Basic epidemiological data (name, age, gender, date, and region of isolation of patients) were recorded for each isolate. We screened for *Shigella* species by using the methods as reported previously [12, 30]. Resultant colonies on the *Salmonella-Shigella* (SS) agar were transferred to our Microbiology Laboratory of Shanxi CDC for further confirmation. API 20E test strips (bioMerieux Vitek; Marcy-1′Etoile, France) and two specific serotyping kits were used to identify all the types and groups of *S. flexneri*. The slide agglutination test was used for serological reactions as reported previously [43].

### Antimicrobial susceptibility testing

The antimicrobial susceptibility of all the *Shigella* isolates (474 *Shigella* strains, including 137 *S. sonnei* and 337 *S. flexneri*) was determined by analyzing the minimum inhibitory concentrations (MICs) of 21 antimicrobial agents, which were tested using the Sensititre semi-automated antimicrobial susceptibility system (TREK Diagnostics, Inc., Westlake, OH, USA) and the Sensititre 96-well plate PRCM2F (Thermo Fisher Scientific Ine, West Sussex, UK) according to the recommendations of the Clinical and Laboratory Standards Institute (CLSI, 2019) [44], as described previously [12, 30]. The 21 antimicrobial agents included CAZ, CRO, FEP, CFP, CFZ, FOX, IPM, NIT, PIP, AMP, TIC, TE, TO, GEN, AK, ATM, C, TIM, LEV, NOR, and SXT. The *Escherichia coli* ATCC 25922 was used as quality control.

### Tests for antibiotic-resistance genes and integrons

The genomic DNA of each isolate was extracted and purified using a commercial Bacteria DNA Kit (TIANGEN Biotech, China). Polymerase chain reaction (PCR) assays were performed as reported previously to screen for resistance genes and intergrons, such as *β*-lactamase [27, 38, 45–47], quinolone resistance-determining region (QRDR) [48], plasmid-mediated quinolone resistance (PMQR) [38, 45, 49], and variable regions of classes 1 and 2 integrons [30] (See Additional file 1). The resultant PCR products were sequenced, assembled and edited using the software Seqman (DNAstar Inc., Madison, WI, USA). We assessed the nucleotide sequence similarity by using the BLST from the NCBI GenBank database (http://blast.ncbi.nlm.nih.gov/Blast.cgi).

### Pulsed-field gel electrophoresis (PFGE)

The genetic relationship among the *Shigella* species isolated from Shanxi Province was determined by analyzing 37 *S. sonnei* strains and 38 *S. flexneri* strains by using pulsed-field gel electrophoresis (PFGE) according to the standard protocol for *Shigella* outlined by PulseNet [50]. Macrorestriction patterns and dendrograms were analyzed and constructed using the methods as described previously [12, 30], but with a different position tolerance of 1.5%.

### Statistical analysis

Statistical analysis was performed using Chi-square test by using SPSS statistical package v.19.0 (SPSS Inc., Chicago, IL). We compared the antibiotic resistance rates between the ages, gender, serotypes, and locations of the patients. A *P* value of < 0.05 was considered statistically significant.

## Additional file

Additional file 1: **Table S1.** Primers for the PCR detection of antimicrobial-resistance determinants used in this study. (PDF 165 kb)

## Data Availability

The datasets used and analyzed during the current study are available from corresponding author on reasonable request.

## Abbreviations

AK: Amikacin
AMP: Ampicillin
ATM: Aztreonam
C: Chloramphenicol
CAZ: Ceftazidime
CFP: Cefoperazone
CFZ: Cefazolin
CLSI: Clinical and Laboratory Standards Institute
CRO: Ceftriaxone
FEP: Cefepime
FOX: Cefoxitin
GN: Gentamicin
IMP: Imipenem
LEV: Levofloxacin
MDR: Multidrug resistance
MIC: Minimum inhibitory concentration
NARMS: National antimicrobial resistance monitoring system
NIT: Nitrofurantoin
NOR: Norfloxacin
PCR: Polymerase chain reaction
PFGE: Pulsed-field gel electrophoresis
PIP: Piperacillin
PMQR: Plasmid-mediated quinolone resistance
QRDR: Quinolone resistance-determining region
SXT: Trimethoprim/sulfamethoxazole
TE: Tetracycline
TIC: Ticarcillin
TIM: Ticarcillin/clavulanic acid
TO: Tobramycin
