# Supplementary material for: Antimicrobial resistance and genetic characterization of Shigella spp. in Shanxi Province, China, during 2006–2016
Source: BMC Microbiol. 2019 May 29;19:116. doi: 10.1186/s12866-019-1495-6 (PMC6542020; doi:10.1186/s12866-019-1495-6)
Supplement: Supplementary file 1 — Table S1. Primers for the PCR detection of antimicrobial-resistance determinants used in this study. (PDF 165 kb) [file 12866_2019_1495_MOESM1_ESM.pdf]

Table 1 Primers for the PCR detection of antimicrobial-resistance determinants used in this study.

Target	Primer sequence(5'-3')	Amplicon size(bp)	Reference
bla$_{\text{CTX-M-1}}$ group	F: GGTTAAAAAATCACTGCGTC<br>R: TTACAAACCGTCGGTGACGA	873	(17)
bla$_{\text{CTX-M-9}}$ group	F: AGAGTGCAACGGATGATG<br>R: CCAGTTACAGCCCTTCGG	868	(17)
bla$_{\text{CTX-M-2/8/25}}$ group	F: ACCGAGCCSACGCTCAA<br>R: CCGCTGCCGGTTTTATC	221	(17)
bla$_{\text{TEM}}$	F: ATGAGTATTCAACATTTCCG<br>R: CCAATGCTTAATCAGTGAGG	1080	(21)
bla$_{\text{OXA}}$	F: ATTAAGCCCTTTACCAAACCA<br>R: AAGGGTTGGGCGATTTTGCCA	890	(22)
bla$_{\text{VIM}}$	F: AGTGGTGAGTATCCGACAG<br>R: ATGAAAGTGCGTGGAGAC	509	(24)
bla$_{\text{CMY}}$	F: GTGGTGGATGCCAGCATCC<br>R: GGTCGAGCCGGTCTTGTTGAA	915	(21)
bla$_{\text{SHV}}$	F: TTATCTCCCTGTTAGCCACC<br>R: GATTTGCTGATTTCGCTCGG	795	(21)
IntI1	F: ACATGTGATGGCGACGCACGA<br>R: ATTTCTGTCCTGGCTGGCGA	569	(20)
*IntI*2	F: CACGGATATGCGACAAAAAGGT<br>R: GTAGCAAACGAGTGACGAAATG	789	(20)
Class 1 integron variable region	hep58: TCATGGCTTGTTATGACTGT<br>hep59: GTAGGGCTTATTATGCACGC	Variable	(17)
Class 2 integron variable region	hep74: CGGGATCCCGGACGGCATGCACG ATTTGTA<br>hep51: GATGCCATCGCAAGTACGAG	Variable	(16)
gyrA	F: TACACCGGTCAACATTGAGG	648	(25)

	R: TTAATGATTGCCGCCGTCGG		
gyrB	F: TGAAATGACCCGCCGTAAAGG<br>R:GCTGTGATAACGCAGTTTGTCCGGG	309	(25)
parC	F: GTACGTGATCATGGACCGTG<br>R: TTCGGCTGGTCGATTAATGC	531	(25)
parE	F: ATGCGTGCGGCTAAAAAAGTG<br>R: TCGTCGCTGTCAGGATCGATAC	290	(25)
qnrA	F: ATTTCTCACGCCAGGATTTG<br>R: GATCGGCAAAGGTYAGGTCA	516	(26)
qnrB	F: GATCGTGAAAGCCAGAAAGG<br>R: ACGAYGCCTGGTAGTTGTCC	469	(26)
qnrD	F: CGAGATCAATTTACGGGGAATA<br>R: AACAAGCTGAAGCGCCTG	656	(21)
qnrS	F: ACGACATTCGTCAACTGCAA<br>R: TAAATTGGCACCCTGTAGGC	417	(26)
aac(6')-Ib-cr	F: GCAACGCAAAAACAAAGTTAGG<br>R: GTGTTTGAACCATGTACA	560	(23)
